# Dopaminergic-Like Neurons Derived from Oral Mucosa Stem Cells by Developmental Cues Improve Symptoms in the Hemi-Parkinsonian Rat Model

**DOI:** 10.1371/journal.pone.0100445

**Published:** 2014-06-19

**Authors:** Javier Ganz, Ina Arie, Sigal Buch, Tali Ben Zur, Yael Barhum, Sammy Pour, Shareef Araidy, Sandu Pitaru, Daniel Offen

**Affiliations:** 1 Neurosciences Laboratory, Felsenstein Medical Research Center-Rabin Medical Center, Tel Aviv University, Tel Aviv, Israel; 2 Oral Biology Dept., School of Dental Medicine, Faculty of Medicine, Tel Aviv University, Tel Aviv, Israel; 3 Oral & Maxillofacial Dept., Baruch Padeh Medical Center, Poria, Lower Galilee, Israel; National Institutes of Health, United States of America

## Abstract

Achieving safe and readily accessible sources for cell replacement therapy in Parkinson’s disease (PD) is still a challenging unresolved issue. Recently, a primitive neural crest stem cell population (hOMSC) was isolated from the adult human oral mucosa and characterized *in vitro* and *in vivo*. In this study we assessed hOMSC ability to differentiate into dopamine-secreting cells with a neuronal-dopaminergic phenotype *in vitro* in response to dopaminergic developmental cues and tested their therapeutic potential in the hemi-Parkinsonian rat model. We found that hOMSC express constitutively a repertoire of neuronal and dopaminergic markers and pivotal transcription factors. Soluble developmental factors induced a reproducible neuronal-like morphology in the majority of hOMSC, downregulated stem cells markers, upregulated the expression of the neuronal and dopaminergic markers that resulted in dopamine release capabilities. Transplantation of these dopaminergic-induced hOMSC into the striatum of hemi-Parkinsonian rats improved their behavioral deficits as determined by amphetamine-induced rotational behavior, motor asymmetry and motor coordination tests. Human TH expressing cells and increased levels of dopamine in the transplanted hemispheres were observed 10 weeks after transplantation. These results demonstrate for the first time that soluble factors involved in the development of DA neurons, induced a DA phenotype in hOMSC *in vitro* that significantly improved the motor function of hemiparkinsonian rats. Based on their neural-related origin, their niche accessibility by minimal-invasive procedures and their propensity for DA differentiation, hOMSC emerge as an attractive tool for autologous cell replacement therapy in PD.

## Introduction

Parkinson’s disease (PD) is characterized by the selective loss of a specific subset of brain neuronal cells - A9 dopaminergic neurons located in pars compacta of the substantia nigra. Loss of these neurons results in reduced dopaminergic secretion in the striatum and consequent motor dysfunction and cognitive insufficiency in advanced cases of the disease. Even though treatment with the drug L-3,4-dihydoroxyphenylalanine (L-dopa) improves motor function, in the long term the drug effect decreases. Replacement of lost dopaminergic (DA) neurons by postmitotic dopaminergic neurons derived from the fetal ventral mesencephalon demonstrated improvement of motor symptoms in part of PD patients [1]–[5]. Several obstacles have prevented the widespread application of these therapeutic modality in PD therapy [6]. Even though the achievements of these studies were limited, they provided proof of principle for cell replacement therapy in PD. The progress made in stem cell (SC) biology has enabled the development of new approaches aimed at coaxing various SC to differentiate into DA neurons. These include genetic manipulation and exposure to a variety of morphogenetic factors or chemical compounds. A myriad of SC populations have been explored in the search for new DA neuron sources including embryonic [7], mesenchymal [8], neural progenitors [9], induced pluripotent SC (iPS) [10] as well as the newly reported induced neuronal cells [11] which are generated directly by fibroblast reprogramming.

To achieve clinical translation, safe and readily accessible sources of SC endowed with a high propensity to differentiate into DA neurons are being intensively sought. In this regard, autologous adult SC are a safe alternative devoid of the immunologic, ethical and safety concerns that might be associated with embryonic, fetal and iPS cells. The lamina propria of the adult oral mucosa is a readily accessible site. The embryonic origin of its cell population is from the neural crest (NC) that develops in the mesencephalic region. The NC is a transient neuroectodermal structure that develops from the neural plate of the vertebrate embryo. During its embryonic existence, the NC gives rise to migratory multipotent SC (NCSC) characterized by the expression of markers as p75, Snail2, Sox10, Twist and Notch 1 [12]–[14]. NCSC colonize various primordial tissues, where they differentiate into neural lineages and lineages of a mesenchymal phenotype termed ectomesenchyme or mesectoderm [15], [16]. Some of these NCSC remain in a relative undifferentiated state in the adult with a propensity for neural differentiation *in vitro* even in tissues of mesenchymal origin such as dermis and bone marrow [17], [18]. However, the frequency of these cells is low, reaching levels below 1% of the total population [18]. Recent data indicate that oral-facial adult tissues comprise higher numbers of NCSC [12], [14], [15], [18]. Marynka-Kalmani et al. have recently isolated from the lamina propria of the adult human oral mucosa a NCSC-like population that was termed human oral mucosa derived-stem cells (hOMSC) (14). Forty to 60% of hOMSC are positive for the pluripotency associated transcription factors Oct4, Nanog and Sox2. Sixty to 70% of hOMSC express constitutively nestin, β-III tubulin and FoxA2 (14). FoxA2 has been recently identified as a pivotal transcription factor in the specification and final differentiation of the DA phenotype [19], [20] and the differentiation of human ESC into DA neurons [21]. Based on this knowledge we hypothesized that soluble factors involved in the development of the DA-neuron phenotype might coax the hOMSC population to differentiate along this lineage. In the present study, we describe the differentiation of hOMSC into DA-like neurons by developmental soluble factors, which improve motor parameters associated with a rat PD model following their intra-striatal transplantation.

## Materials and Methods

All the protocols used in this manuscript were approved by the Institutional Helsinki Committee at the Baruch Padeh Medical Center, Poria and Tel Aviv University, Israel.

### hOMSC Culture

hOMSC were obtained from oral mucosa biopsies from four different healthy donors 20, 24, 29 and 35 years old after obtaining written informed consent from the donors and the approval of the Institutional Helsinki Committee at the Baruch Padeh Medical Center, Poria, Israel. hOMSC were generated and expanded in medium consisting of low-glucose Dulbecco’s modified Eagle’s medium supplemented with 100 µg/ml streptomycin, 100 U/ml penicillin, (Biological Industries, Beit-Haemek, Israel), glutamine 2 mM (Invitrogen, Carlsbad, CA, USA) and 10% fetal calf serum (FCS) (Gibco) as described by Marynka-Kalmani et al 2010 [13]. Briefly, biopsies were incubated at 4°C overnight in dispase (Sigma, Israel). Then, the epithelial layer was separated from the lamina propria and the last was minced into pieces of about 0.5 mm^3^. The pieces were placed on the floor of 35 mm culture dishes (Nunc). The above mentioned expansion medium was added gently to the explants to allow their attachment to the floor of the dish. Cells that emigrated from the explant to the culture dishes were harvested with 0.25% Trypsin (Biological Industries, Beit-Haemek, Israel) and seeded at a cell density of 4×10^4^ cells/1 cm^2^. Cells were passaged at 70–80% confluence. hOMSC at passages 4–20 were used in the below described experiments.

### Dopaminergic Differentiation of hOMSC

hOMSC were cultured in serum free medium supplemented with N2 (GIBCO), basic fibroblast growth factor 2 (bFGF) (R&D Systems, Minneapolis, MN, USA) and epidermal growth factor (EGF) (R&D Systems, Minneapolis, MN, USA), each at a final concentration of 20 ng/ml for 48 hr. Thereafter, the cultures were incubated for 15 days in Neurobasal medium supplemented with 0.5% B27, 250 ng/mL Sonic Hedgehog (Shh), 100 ng/mL of Wnt-1 and FGF-8, 50 ng/mL of BDNF and bFGF and 200 µM of ascorbic acid. All factors were obtained from Peprotech Asia unless otherwise specified. hOMSC cultures maintained only in serum free medium, served as controls for the experiments described below. hOMSC were differentiated into osteoblastic-like cells described elsewhere [13], serving as differentiation controls for immunofluorescence studies.

### Midbrain Mouse Primary Culture

Mouse mesencephalic primary cultures were generated as described elsewhere [22] using four days old mice. 250,000 cells were seeded in 24 well plates and examined for dopaminergic neurons markers analysis after fixation with 4% paraformaldehyde (PFA).

### Mouse Embryonic Fibroblasts

Mouse embryonic fibroblasts (MEF) were isolated from mice embryos on embryonic day 14.5. Internal organs, eyes, and spinal cord were carefully removed as described elsewhere [23]. MEFs were passaged at least three times before seeded in 24 well plates, fixed with 4% PFA and examined for dopaminergic neurons markers.

### Real-time PCR

Total RNA from four donors were isolated by TRI reagent (Invitrogen, Carlsbad, CA, USA) according to the supplier’s recommendations. 2 µg of RNA was used for reverse transcription performed with random primers and SuperScriptIII (Invitrogen, Carlsbad, CA, USA). Real-time PCR of the genes of interest was performed in an ABI Prism 7700 sequence detection system (Applied Biosystems) by using PlatinumR SYBRR Green qPCR SuperMix UDG with ROX (Invitrogen, Carlsbad, CA, USA). PCR amplification was performed by 40 cycles (program: 2 min at 50°C; 2 min at 95°C; 40 repeats of 15 s at 95°C and 30 s at 60°C). Data were quantified by using the ΔΔCt method, normalized to glyceraldehyde-3-phosphate dehydrogenase (GAPDH) housekeeping gene and using the ΔCt of undifferentiated cultures as baseline. Data are presented as the mean ± standard deviation (SD) change from the baseline. Primers’ sequences used for RT-PCR analysis of pluripotency, neuronal and dopaminergic markers are shown in Table S1.

### Immunofluorescence

Cells obtained from two donors (24 and 35 years old) were fixed in 4% PFA-PBS and pre-incubated for 60 min in blocking solution (5% goat serum, 1% BSA, 0.05% Triton-X in PBS). Primary antibodies were diluted in the blocking solution and applied overnight at 4°C. The following primary antibodies were used, β-III-Tubulin (1∶200, AB7751, abcam), Map2 (1∶200, AB5622, Chemicon, Temecula, CA, USA), synapsin (1∶200, AB1543, Millipore, Billerica, MA, USA), tyrosine hydroxylase (1∶200, B152, Sigma, Israel), Lmx1A (1∶200, MB369, Chemicon, Temecula, CA, USA), Pitx3 (1∶200, AB5722, Chemicon, Temecula, CA, USA) and Nurr1 (1∶200, AB5778, Millipore, Billerica, MA, USA). Primary antibodies were detected with fluorescent-labeled secondary antibodies Alexa 488 and 568 (1∶500, Molecular Probes) for 1 h at room temperature. Nuclear DNA was stained using 4,6-diamino-2-phenylindole (DAPI) (1∶1000, Sigma, Israel). Cells that were incubated overnight with the blocking solution and then incubated only with the secondary antibodies served as negative controls and for determining the photography exposure settings (Fig. S1). Midbrain primary cultures served as positive controls for dopaminergic markers; mouse embryonic fibroblasts and hOMSC-derived osteoblastic cultures (Marynka-Kalmani et al.) served as negative controls. To quantify the number of cells positive for dopaminergic or neuronal markers and the number of cells co-expressing both types of markers captures of immunostained cultures derived from 2 different donors were obtained with a fluorescence Olympus IX70-S8F2 microscope (excitation wavelength, 330–385 nm; barrier filter, 420 nm) and a U-MNU filter cube (Olympus, Center Valley, PA). Cell counts for single or double-labeled specimens were performed on three non-overlapping randomly selected fields in cultures obtained from each of the donors. The results were expressed as Mean ± SEM.

### Western Blot

Cells were lysed in buffer containing PBS, 1%SDS, complete protease inhibitor (Sigma, Israel) and loaded into SDS-PAGE 12%. Three cell cultures from each condition were used as biological replicates. Gels were transferred using liquid transference (300 mA, 1.15 hs) and membranes were blocked using PBS buffer 5% milk for 2 hours at room temperature. The membranes were probed with anti-TH (Sigma, Israel), anti-Pitx3 (Chemicon, Temecula, CA, USA), anti-Lmx1A (MB369, Chemicon, Temecula, CA, USA), anti-Nurr1 (AB5778, Millipore, Billerica, MA, USA), anti-emerin (06-1052, Millipore, Billerica, MA, USA) and anti-actin (Chemicon, Temecula, CA, USA), followed by IRDye (680, 800 nm) conjugated secondary antibody (LI-COR, Nebraska, USA). The membranes were analyzed and bands quantified with Odyssey infrared imaging systems (LI-COR, Nebraska, USA).

### Dopamine Quantification

Cellular dopamine release determination was performed by reverse-phase HPLC, using three independent cell cultures for each condition. Media of differentiated and naïve cell cultures were replaced first with HBSS buffer for 35 minutes at 37°C. Then, the HBSS buffer was replaced with new HBSS buffer supplemented with 56 mM KCl to induce membrane depolarization for another 35 minutes at 37°C. The supernatant was collected and stabilized with 4 mM sodium meta-bisulfite (Sigma, Israel) and 1 mM EDTA (Sigma, Israel), and stored at −80°C. Dopamine was extracted by aluminum adsorption and analyzed by injection (20 µl) into a HPLC system (Waters, Milford, MA, USA) equipped with a C18 reverse phase, 3 µm LUNA column (100 mm×2 mm; Phenomenex, Torrance, CA, USA). Samples were eluted by 25 mM NaH_2_PO_4_, 50 mM Na-citrate, 0.03 mM EDTA, 10 mM diethylamine HCl, and 2.2 mm sodium octyl sulfate (pH 3.2), 30 ml/L methanol and 22 ml/L dimethylacetamide at a flow rate of 0.4 ml/min. Dopamine peak was determined by electrochemical detection at a potential of 0.6 V. The dopamine content was calculated by extrapolating the peak area from a standard curve (range 1–200 pg of dopamine) constructed under the same conditions during each run by the Maxima Workstation (Waters). Dopamine concentration is expressed as Mean ± SEM of dopamine content/1 million cells.

### The Hemiparkinsonian Rat Model, Cell Transplantation and Behavioral Analysis

Eight-week-old male Sprague-Dawley rats (Harlan, Israel), weighing 230–250 g, were maintained under 12-hour-light/12-hour-dark conditions and grown in individually ventilated cages (IVC) with ad libitum access to food and water. All experimental protocols were approved by the Tel Aviv University Committee of Animal Use for Research and Education. Thirty animals were divided into 3 groups, 10 animals in each. Animals in the first, second and third group were allocated for transplantation with hOMSC subjected to dopaminergic differentiation, naïve hOMSC and saline, respectively. The animals were anesthetized with ketamine and xylazine (60 mg/kg and 10 mg/kg, respectively). The DA denervation was induced by stereotactic injections of 6 µl of 6OHDA (at a concentration of 2.5 mg/ml in normal saline supplemented with 0.02% ascorbic acid into the *medial forebrain bundle* (MFB) (anterior −4.0 mm; lateral −1.3 mm; ventral −7.7 mm, as determined from the bregma and the skull surface). Only animals that exhibited an amphetamine-induced rotational behavior (>2 rotations/min) were selected for further experimentation. DA-differentiated and naïve hOMSC were labeled with PKH26 (Sigma, Israel), harvested and resuspended at a concentration of 1×10^5^ cells/µl of saline, and maintained on ice until transplantation. Four µl of cell suspension were injected at each of two different coordinates (AP −0.8 ML −4.2 DV −5.5, AP 0.0 ML −2.8 DV −4.6). Tripan blue staining was performed in parallel aliquots to ascertain 99% cell viability. The following motor function assays were performed:

- Amphetamine induced-rotations test: The effect of subcutaneous amphetamine injections (2.5 mg/kg, Sigma, Israel) on the number of rotations was measured for 60 min using an automated Rotameter device (San Diego Instruments, CA, USA). The net ipsilateral rotations were measured 2 weeks after 6OHDA injection. This time point was considered time 0. Only rats with two or more rotations/min were transplanted 24 hours after the rotation test as described above. The effect of cell transplantation on animal rotations was determined at 1, 4 and 8 weeks post-transplantation.
- Cylinder test: Motor asymmetry was measured by the cylinder test three weeks after cell transplantation as previously described elsewhere[24]–[26] Briefly, the number of wall contacts with each forelimb when rearing in at least 15 rearing cycles was computed. Animals that did not meet this criterion were excluded from this assay. The cylinder test score was determined as follows: (use of the affected forepaw (contralateral) – intact forepaw (ipsilateral)/total (contralateral + ipsilateral + both).
- Rotarod test: Motor activity was assessed by the rotarod test using the San Diego Instrument, Rotor-Rod (San Diego Instruments, CA, USA) (0–25 RPM). Hemiparkinsonian animals were tested 2 days before cell transplantation to determine baseline values. Then, they were tested 2 days after the amphetamine induced rotation test, that is 9, 16 and 30 days after cell transplantation. Each test consisted of 3 consecutive measurements of the time each animal remained on the rod without falling. The results were expressed as the percentage of change from the baseline.

### Brain Tissue Analysis

The brains of transplanted and control (saline-treated) animals were analyzed for: i) the identification of the transplanted cells within the striatum and for their capacity to express tyrosine hydroxylase by immunofluorescence; ii) the expression of tyrosine hydroxylase within the *substantia nigra* by immunohistochemistry; and iii) the level of dopamine in the healthy and affected hemispheres by HPLC. Four animals of each group were used for the immunochemistry and immunofluorescence assessment.

#### Tyrosine hydroxylase expression

Rats were anesthetized and perfused with PFA 4%. Brains were dissected, embedded in OCT and sectioned (10 µm) using a cryostat. Antigen retrieval was performed by boiling the slides in 10 Mm citrate buffer for 10 min. Sections were blocked in 5% goat serum, 1% BSA, 0.05% Triton-X in PBS for 2 hr and incubated with primary antibodies overnight at 4°C. For immunofluorescence, sections were incubated with conjugated secondary antibodies.For immunohistochemistry the DAB (3-3′ diaminobenzidine) peroxidase kit (Vector Laboratories, Burlingame, CA, USA) was used to visualize the primary antibody according to the manufacturer’s instructions. Sections incubated overnight with blocking solution and then only with the secondary antibodies or with DAB, served as negative controls and for determining the photography exposure settings (supplemental figure S1). Sections from the same animals were used for immunochemistry and immunofluorescence. Six slides/animal were used for immunodetection.

#### Dopamine content

To determine dopamine levels, brains of 4 animals of each of the 3 groups were used. Brains of rats sacrificed by CO_2_, were quickly removed and kept on ice. The control and treated hemispheres were separated, homogenized in ice cold 0.1 perchloric acid, centrifuged and supernatants were analyzed for dopamine content by HPLC as described above.

### Statistical Analysis

Results are expressed as mean ± SEM. All analyses were performed using SPSS version 19 software. Differences between two groups were statistically analyzed by T test, while One Way ANOVA analyzed comparisons between three groups. For the cell transplantation *in vivo* experiment, Two Way ANOVA was performed. Tukeys multiple comparison posthoc test was used to evidence specific differences between groups. Significance levels are as follows: *p<0.05, **p<0.01, ***p<0.001.

## Results

### Dopaminergic Differentiation of Naïve Homsc

For the purpose of this investigation hOMSC were generated from the lamina propria of the oral mucosa of 4 young healthy donors (See Materials and Methods) and analyzed by flow cytometry to verify that their marker profile fulfills the criteria determined for this hOMSC population. These criteria previously published by us [13] were determined by characterizing and determining the range of the percentage of positive cells for a number of markers. The data is based on hOMSC separate cultures generated from 25 different donors. According to these criteria, 60%–70% of hOMSC cultures at 10–15 cumulative population doublings express Oct4, Sox2 and nestin and more than 95% of the cells are positive for CD29, CD73 and CD105.

**Figure 1:**
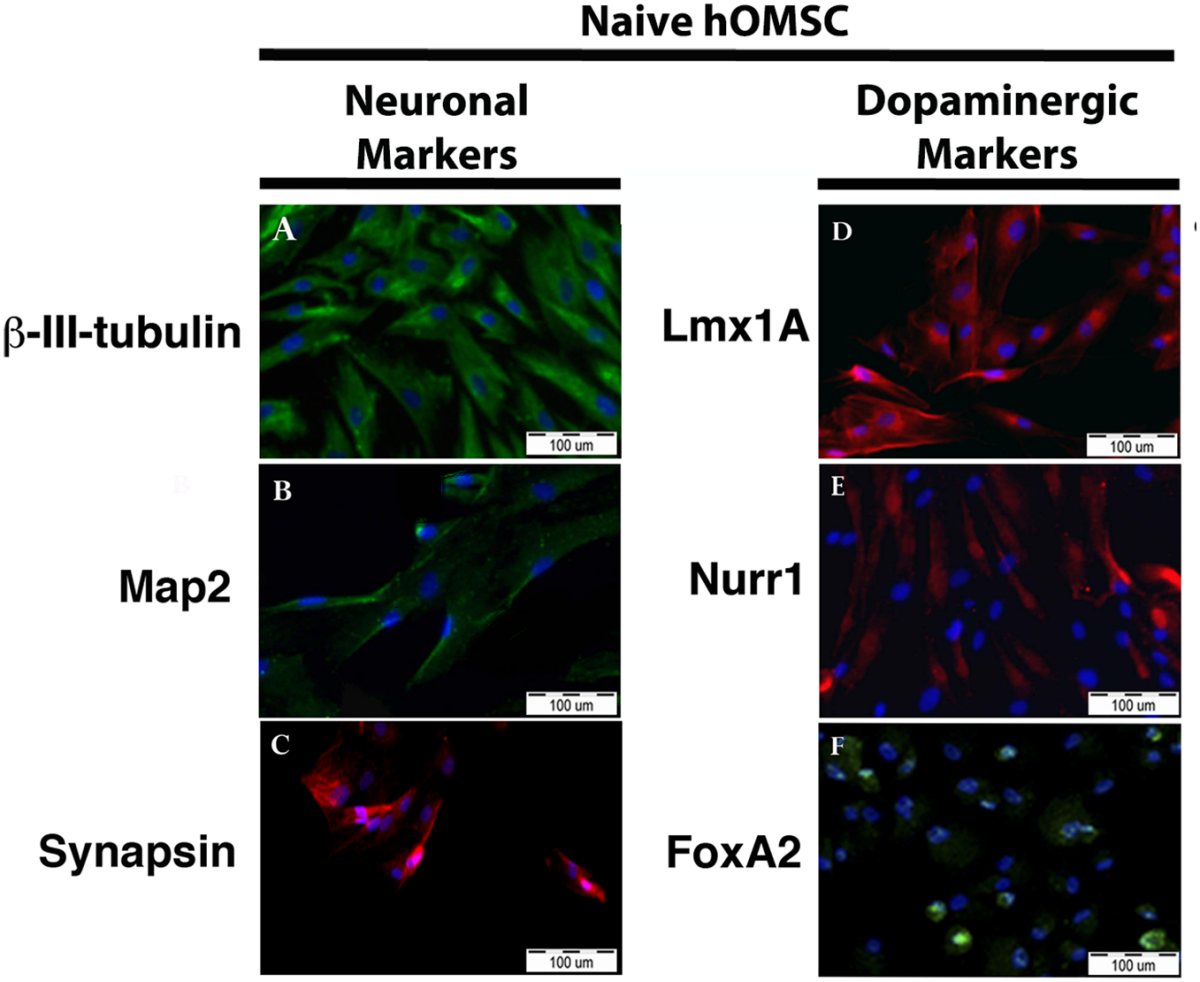
Naive hOMSC express constitutively neuronal and dopaminergic markers. Immunofluorescence of neuronal (A–C) and dopaminergic (D–F) markers in naïve hOMSC, scale bars 100 µM.

**Figure 2:**
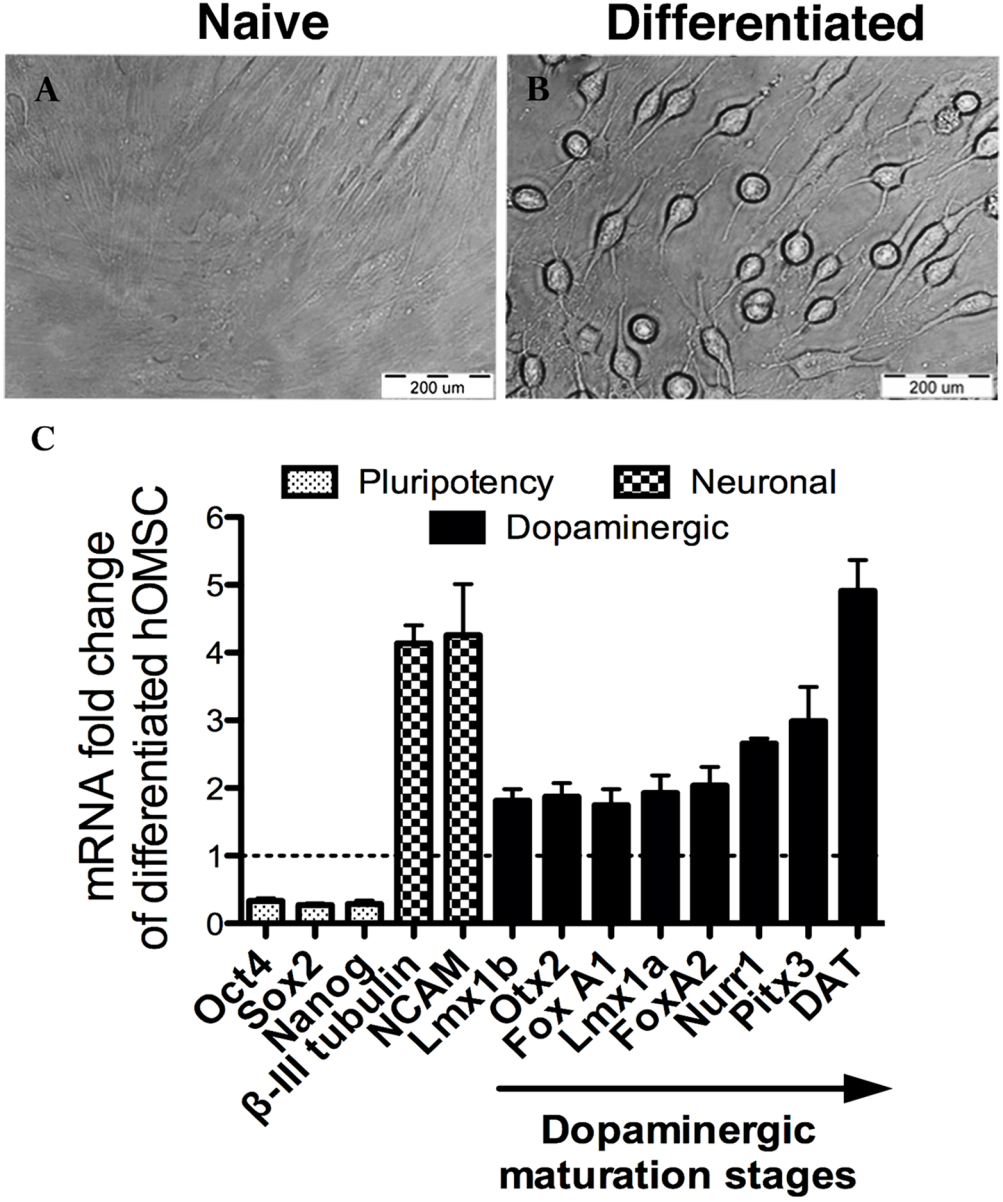
hOMSC developed neuronal-like morphology and gene expression after DA differentiation protocol. Bright field microscopy of hOMSC cultured in serum free medium for 17 days and of hOMSC-DA (A–B), scale bar 200 µM. Real time PCR analysis of differentiated hOMSC-DA from four different donors (C), showing a reduction in the expression of Nanog, Oct4 and Sox2 and a concomitant increased expression of the neuronal markers β-III tubulin and NCAM1 compared to the naïve hOMSC which served as baseline (relative value = 1). Differentiated hOMSC also showed increased expression of genes related to dopaminergic differentiation and mature dopaminergic neurons. Data is expressed as Mean ± SEM. Significance levels: *p<0.05, **p<0.01, ***p<0.001.

In the present study it was found that naïve hOMSC fulfilling these criteria and cultured in expansion medium (low glucose DMEM+10% FCS) expressed constitutively the neural markers β- III tubulin (TUJ1), MAP2 and synapsin as well as the characteristic dopaminergic neurons transcription factors FoxA2, Lmx1A and Nurr1 (Fig. 1). Whereas FoxA2 was located mainly in the nuclei, Lmx1A and Nurr1 were identified mainly in the cytoplasm (Fig. 1D–F). Dopaminergic differentiation of naïve hOMSC into dopaminergic-like cells (hOMSC-DA) was achieved by subjecting naïve hOMSC cultures to a cocktail of growth and differentiation factors found to be involved in neuronal growth, dopaminergic specification and maturation *in vivo* or known to induce the differentiation of ESC along the dopaminergic lineage *in vitro*. Morphological changes in the spindle shape of naïve hOMSC appeared by the 3rd day of differentiation and peaked by day 17^th^ when the majority of the cells assumed a bipolar or multipolar appearance with cellular processes extending from cell bodies (Fig. 2A). At this stage, cultures were analyzed for the expression of dopaminergic markers by RT-PCR, quantitative immunofluorescence and western blots.

RT-PCR analysis of differentiated cultures derived from 4 different donors revealed a significant decrease in the expression of the pluripotency markers Oct4, Sox2 and Nanog at the end of the differentiation processes as well as an increase in the neuronal marker β-III tubulin and NCAM1 (Fig. 2B). The transcript levels for the dopaminergic markers Otx2, Lmx1A/B, FoxA1, FoxA2, Nurr1, Pitx3, and DAT, were significantly increased compared to baseline (Fig. 2B). RNA-transcripts corresponding to the late stages of dopaminergic differentiation exhibited the highest increase in expression compared to baseline (Fig. 2B).

**Figure 3:**
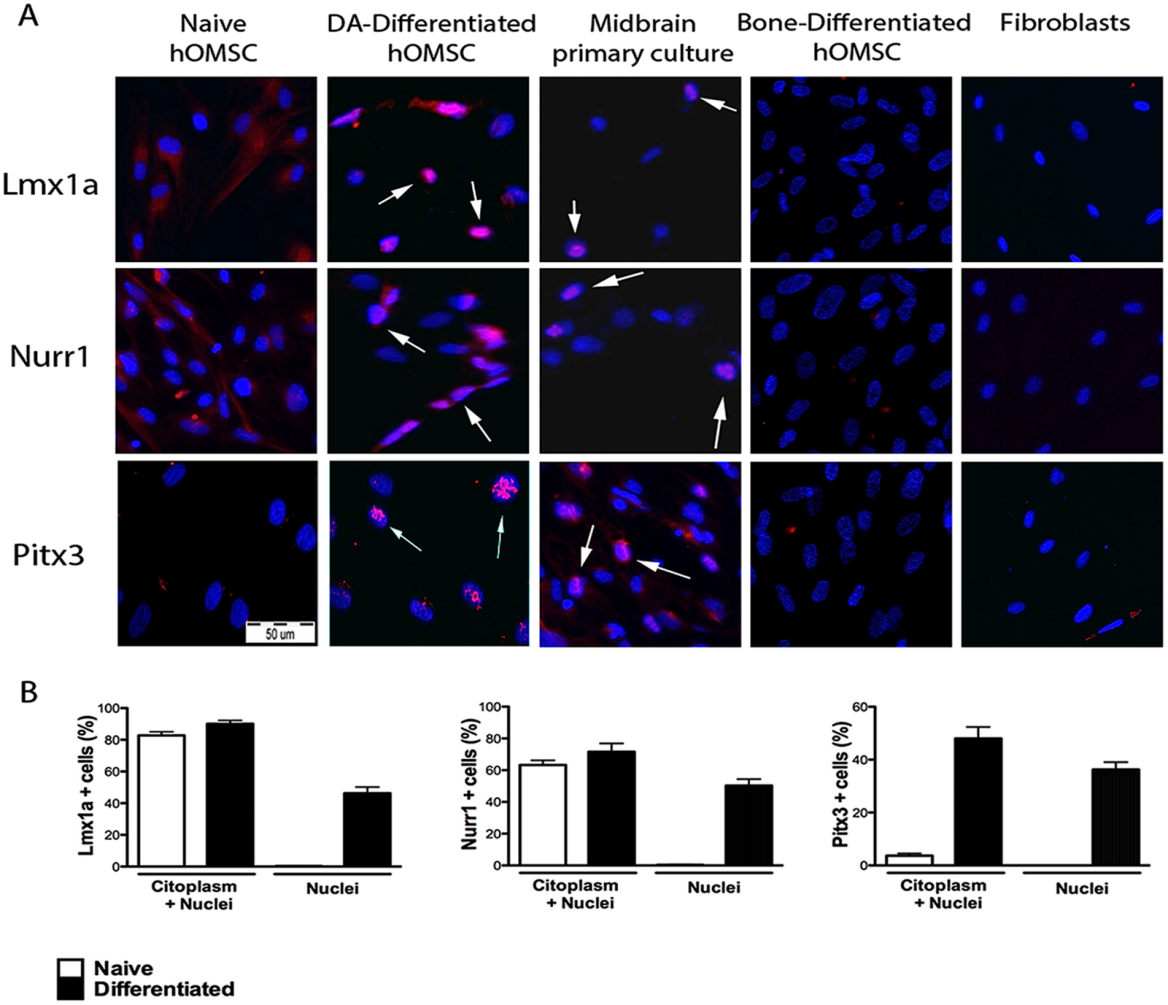
Increased expression and nuclear translocation of dopaminergic transcription factors was observed in hOMSC-DA. Immunofluorescent detection of Pitx3, Lmx1A and Nurr1 evaluated in naïve and differentiated hOMSC, midbrain primary culture, hOMSC-derived osteoblast-like cells and mouse embryonic fibroblasts (A). Arrows point to the nuclear localization of these transcription factors in the hOMSC-differentiated and midbrain DA cells. Scale bar = 50 µM. Quantification of cells expressing the evaluated transcription factors in the whole cell (nuclei+cytoplasm) or only in the nuclei before and after DA-differentiation (B).

Naïve and DA-differentiated hOMSC were evaluated for presence and cellular localization of three transcription factors involved in DA differentiation, Lmx1a, Nurr1 and Pitx3 (Fig. 3). In contrast to the localization of these proteins in the cytoplasm of naïve hOMSC, they were mainly identified in the nuclei of hOMSC-DA (Fig. 3A). In order to verify the specificity of the staining,

western blot analysis of naïve and differentiated hOMSC using the Lmx1A and Nurr1 antibodies revealed specific staining in both cases, whereas fibroblasts lysate failed to show any immunoreactivity (Fig. S2). To finally confirm the staining specificity, mouse midbrain primary cultures, consisting of neurons and glia, served as a positive control demonstrating positive staining in the nuclei of a number of cells. As negative controls, hOMSC differentiated into osteoblast-like cells and mouse fibroblasts were also evaluated and found to be negative (Fig. 3A). Positive cell quantification revealed that: i) Pitx3 was expressed in 3.667%±0.8819 and 48.00%±4.359 of the cells before and after differentiation, respectively; ii) Nurr1 was expressed in 63.33%±2.906 and 71.67%±5.239 of the cells before and after differentiation respectively; and iii) Lmx1a was expressed in 82.77%±2.282 and 90.17%±2.010 of the cells before and after differentiation respectively (Fig. 3B). Analysis of nuclear localization of these markers indicated that naïve cells did not showed any nuclear staining, whereas in DA-differentiated cells Lmx1a, Nurr1 and Pitx3 were observed in the nuclei of 46.33%±3.844, 50.33%±4.055 and 36.30%±2.779 of the cells, respectively (Fig. 3B).

**Figure 4:**
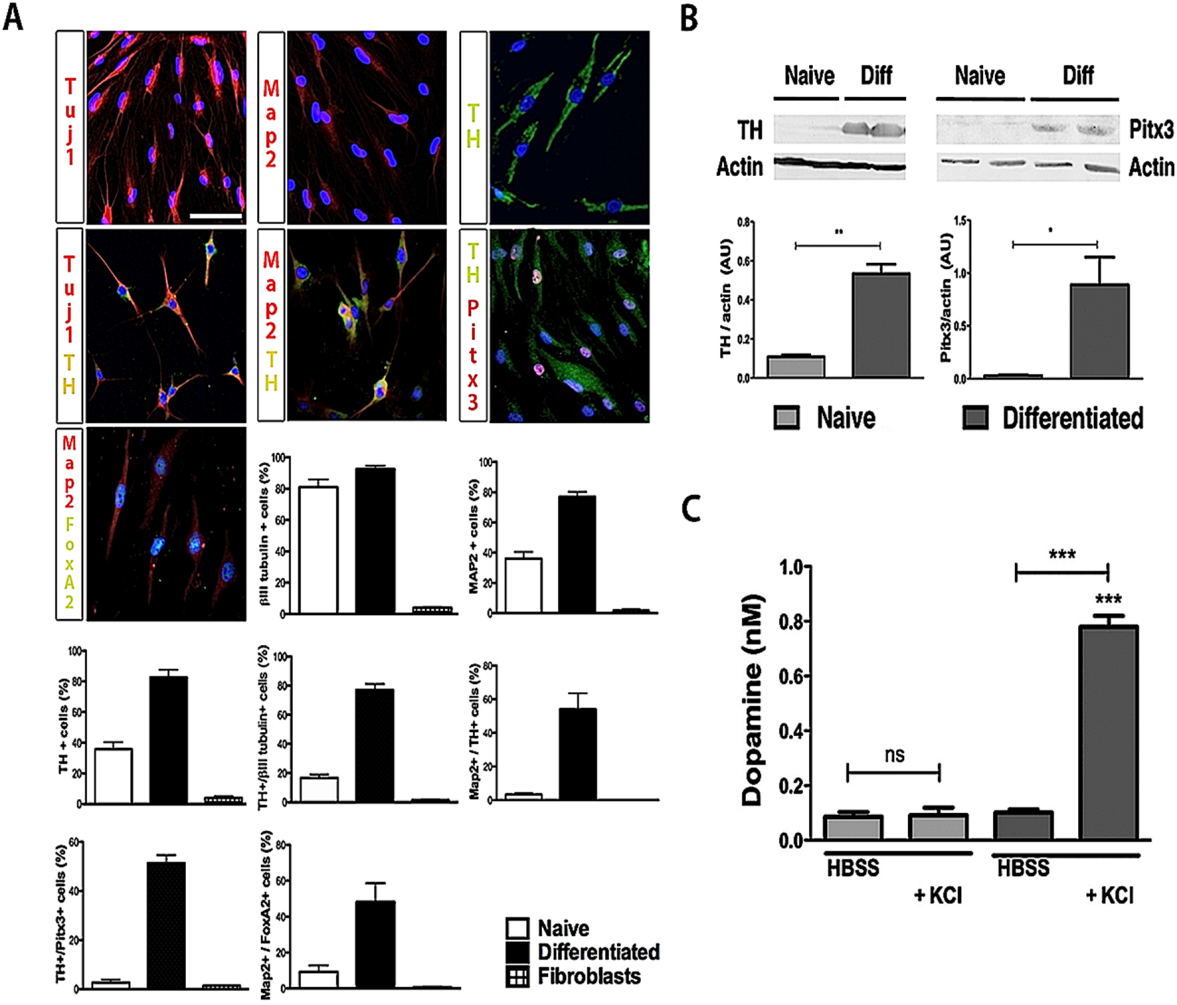
Induced hOMSC show a mature dopaminergic-like phenotype *in vitro*. Immunofluorescence analysis and cell positive counts of neuronal and DA markers in naïve, DA-hOMSC and fibroblasts before and after differentiation, scale bar = 50 µM (A). Complete evaluation of naïve, differentiated hOMSC and fibroblasts marker expression can be seen in supplemental figure S2. Evaluation of Pitx3 and Tyrosine hydroxylase (TH) expression levels by western blot and band densitography, showing increased protein expression after differentiation (B). Medium dopamine content evaluation in naïve or hOMSC-DA in presence or absence of the non-specific membrane depolarizer agent KCl, showing increased levels of dopamine after membrane depolarization of differentiated hOMSC (C). Data is presented as Mean±SEM. Significance levels: *p<0.05, **p<0.01, ***p<0.001.

Immunofluorescence analysis of general neuronal and specific DA markers of naïve and differentiated hOMSC showed that 81.00%±4.933 and 92.67%±2.028 of the cells were positive for βIII-tubulin, respectively; 36.00%±4.583 and 77.00%±3.215 of the cells were positive for MAP2, respectively; and 35.67%±4.702 and 82.67%±4.978 were positive for tyrosine hydroxylase (TH) respectively (Fig. 4A). Double staining analysis for neuronal and DA markers revealed an increase in the number of cells co-expressing both types of markers: the number of cells positive for TH^+^/βIII-tubulin^+^, TH^+^/Map2^+^, TH^+^/Pitx3^+^, and Map2^+^/FoxA2^+^ increased by 4, 9, 8 and 4 fold, respectively after differentiation (Fig. 4A). These data indicate that 83% of the differentiated hOMSC that were positive for β-III tubulin were also positive for tyrosine hydroxylase. Thus, these double-labeled cells represented 77% of the total population of differentiated hOMSC. Complete evaluation of naïve, differentiated hOMSC and fibroblasts regarding neuronal and DA marker expression can be seen in supplemental figure S3.

Western blot analysis confirmed that the protein levels of the end-stage transcription factor Pitx3 and of the characteristic dopaminergic marker TH, were enhanced in the differentiated hOMSC-DA cultures by 5- and 10-fold, respectively, compared to naïve hOMSC control cultures (Fig. 4B). To test to what extent the expression of TH, the rate limiting enzyme in the synthesis of dopamine, is translated into dopamine release, naïve hOMSC and hOMSC-DA were challenged with the non-specific membrane depolarization agent KCl [27] for 30 min and the medium was tested for dopamine content by HPLC. The amount of dopamine released by the hOMSC-DA was by 10 folds higher than that released by naïve hOMSC (0.8 vs. 0.08 nM/1×10^6^ cells/30 min) pointing to the possible therapeutic benefit of hOMSC-DA in PD (Fig. 4C).

### hOMSC-DA Transplantation and Engraftment in the Hemiparkinsonian Rat Model

**Figure 5:**
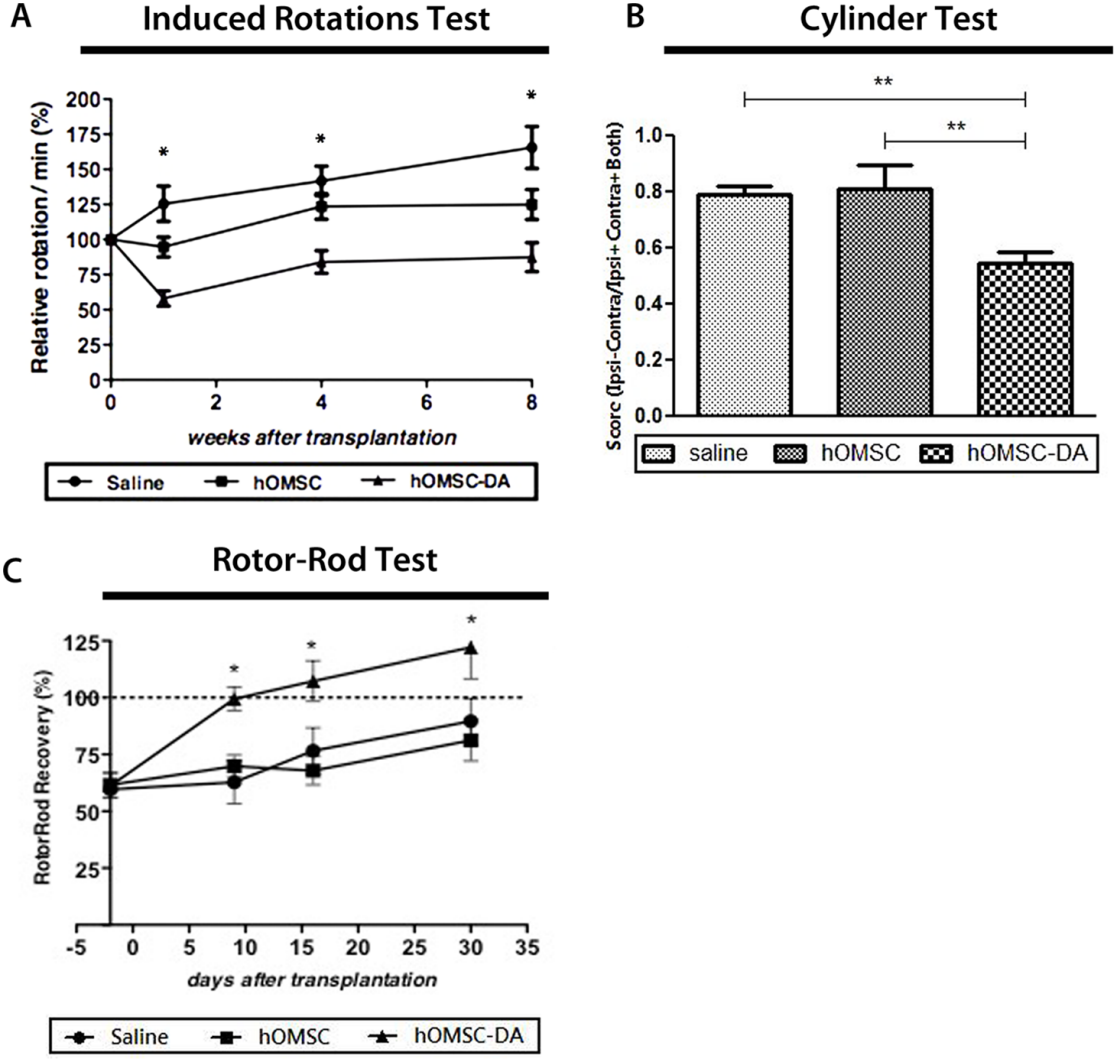
Induced hOMSC-DA show sustained therapeutic effect in a PD rat model after transplantation. Amphetamine-induced rotations (A), rotorod (B) and cylinder (C) test evaluation of 6-OHDA affected rats, following intra-striatal transplantation of saline, naive and hOMSC-DA. Data is presented as Mean±SEM. Significance levels: *p<0.05, **p<0.01, ***p<0.001.

The hemiparkinsonian rat model was generated by inducing a chemical lesion in the nigrostriatal pathway by injecting 6-hydroxy-dopamine (6OHDA) into the medial forebrain bundle. Animals exhibiting nigrostriatal pathway deficit in one hemisphere (see Materials and Methods), rotate in the contralateral direction of the lesioned side after being injected with amphetamine. The number of rotations following amphetamine stimulation of hemiparkinsonian animals indicates the severity of the disease, namely the higher the number of rotations the more severe is the nigrostriatal lesion. hOMSC-DA and naïve hOMSC were labeled with the red-fluorescent dye PKH26 for tracing purposes. Then, diagnosed hemiparkinsonian rats were transplanted at 2 sites of the ipsilateral striatum with either 8×10^5^ hOMSC-DA (n = 9 rats) or with naïve hOMSC (n = 8 rats) or injected with saline (n = 8 rats). A significant reduction of 54%, 40% and 47% in the number of rotations of hOMSC-DA transplanted animals compared to the saline treated group was observed at 1, 4 and 8 weeks post-transplantation, respectively (p<0.05) (Fig. 5A). Even though to a lesser extent, a similar significant reduction in the number of induced rotations was observed in the hOMSC-DA treated rats compared to the naïve hOMSC treated ones (p<0.05). A trend of reduction in the induced rotation was observed in animals treated with naïve hOMSC that reached statistical significance compared to saline-treated animals at week 8 (p<0.05).

Motor asymmetry was assessed by the cylinder test three weeks after transplantation. In this test, the level of motor asymmetry is examined by determining the frequency of use of the ipsilateral (unaffected) and contralateral (affected) forelimbs during rearing and landing in a transparent cylinder. The more frequent use of the ipsilateral forelimb (unaffected) was evident in all the evaluated groups. No differences were observed between saline- or naïve hOMSC-treated rats in this respect (Fig. 5B). hOMSC-DA treated rats showed a significant reduction of 38% in motor asymmetry in comparison to the other two tested groups (p = 0.0009). These data point to an increase in the use of the contralateral forelimb (affected) in the hOMSC-DA transplanted animals compare to the animals in the other two groups.

General motor coordination was assessed by the rotarod test at 9, 16 and 30 days after cell transplantation (Fig. 5C). Motor coordination was assessed by determining the time that an animal can remain without falling on a rotating rod of the rotarod machine. At 1 day after cell transplantation the animals of all 3 groups exhibited an average drop of 40% (p = 0.0086) in the time they remained on the rotarod machine without falling. At 9 days post-transplantation, no significant recovery was observed in the saline or naïve hOMSC treated rats (62.7%±9.4 and 69.8%±4.8 of the values before the administration of 6OHDA, respectively). Animals treated with hOMSC-DA fully recovered, reaching 99.4%±5.1 of their performance before the administration of 6OHDA (Fig. 5C). At 16 and 30 days after transplantation, the animals in all groups displayed an improvement in their performance possibly due to a learning curve. At 30 days post-transplantation, hOMSC-DA treated animals performed by 25% higher than those treated with naïve hOMSC or saline. At this point the test was terminated.

### hOMSC-DA-like Neurons Express TH Ten Weeks after Intra-striatal Transplantation

**Figure 6:**
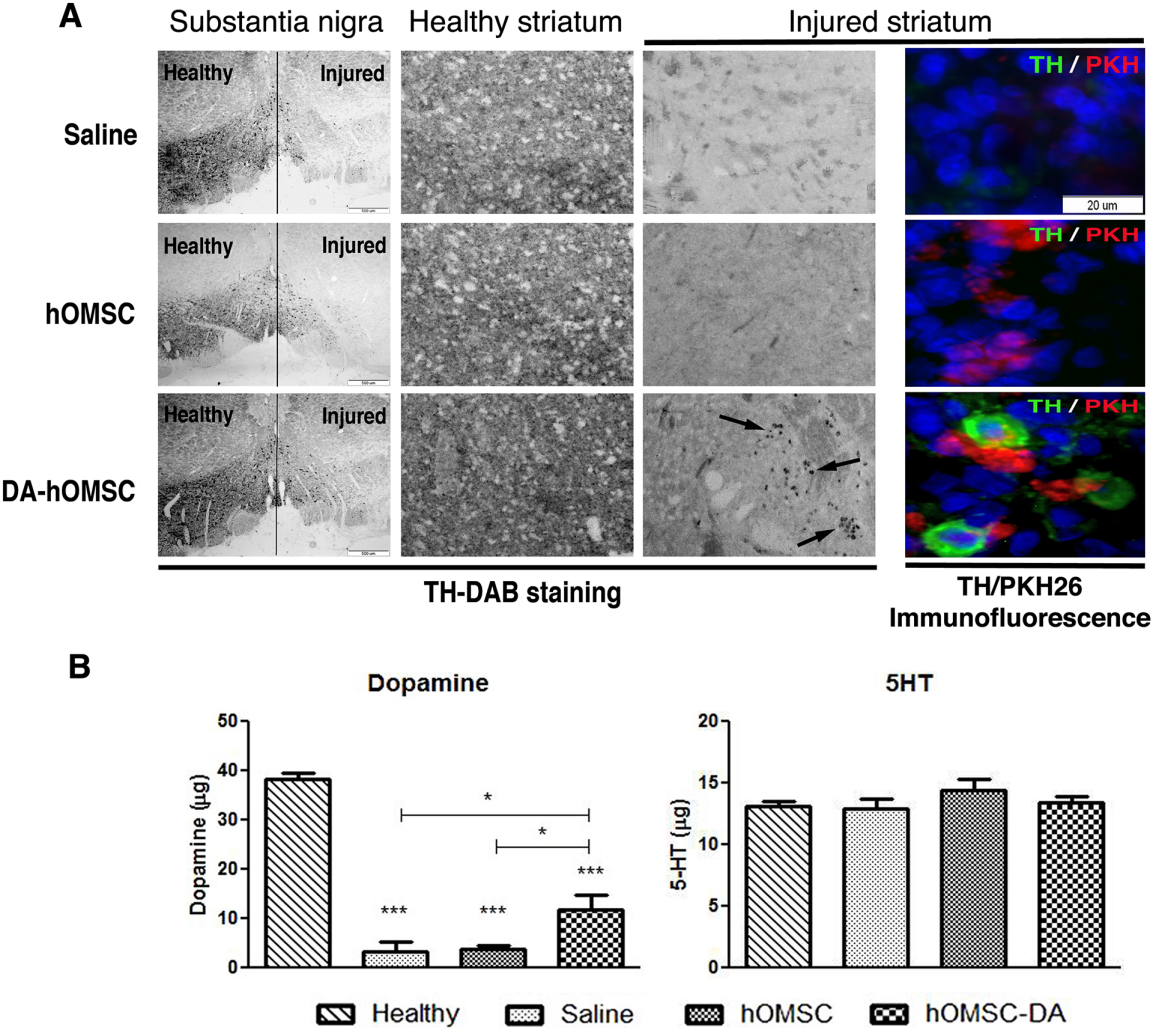
Transplanted cells engrafted the injured hemisphere and increased dopamine levels. Ten weeks post-transplantation TH immunostaining (A) show decreased levels of TH in injured striatum and *substantia nigra* compared to healthy ones. TH striatal expression, analyzed by TH-DAB immunochemistry was detected in hOMSC-DA transplanted hemispheres, while saline or naïve hOMSC transplanted hemispheres showed significant reduced levels (black arrows indicate TH expressing cells). Injured hemispheres transplantated with hOMSC contain PKH26 (red) labeled cells. In hOMSC-DA transplanted hemispheres analyzed by immunofluorescence, TH expression (green) and PKH26 were found in the same cell clusters. Scale bar = 50 µm. (B) Whole hemisphere dopamine and serotonin HPLC quantification from healthy, and 6-OHDA rats treated with saline, naive and hOMSC-DA. Data is presented as Mean±SEM. Significance levels: *p<0.05, **p<0.01, ***p<0.001.

Ten weeks after cell transplantation, rats of the 3 groups were sacrificed. PKH26 positive cells were identified in the striatum of naïve hOMSC and in hOMSC-DA treated animals by immunostaining (Fig. 6A). No tumor formation was detected in any of the implanted sites. Detection of TH by immunofluorescence and immunochemistry revealed strong reactivity in the healthy *substantia nigra* and striatum of the hemispheres that were not injected with 6OHDA in animals of the 3 groups (Fig. 6A). TH was detected to a lower extent in the striatum of 6OHDA-injected hemispheres of animals treated with hOMSC-DA and was barely detected in the striatum of these hemispheres in rats treated with either naïve hOMSC or saline (Fig. 6A). TH expression was significantly reduced in the affected *substantia nigra* of the 6OHDA injected hemispheres of the 3 groups of rats. TH was localized in PKH26 positive cells, indicating that hOMSC-DA cells express TH, while no colocalization was achieved in the other groups (Fig. 6A).

HPLC analysis of dopamine levels in the healthy and injured hemisphere pointed to a reduction in the dopamine levels in all the injured hemispheres compared to those in the healthy ones (11.8±3.0 µg, 3.8±2.2 µg and 3.4±1.8 µg in the hOMSC-DA, naïve hOMSC and saline treated hemispheres, respectively vs. 38.2±3.2 µg in the healthy hemispheres) (Fig. 6B). However, the dopamine level in the hOMSC-DA treated injured hemispheres was 3-fold higher than in the other 2 groups of injured hemispheres. HPLC analysis of serotonin (5HT) revealed similar levels in all the hemispheres (Fig. 6B) indicating that the changes observed in the dopamine levels were specific.

## Discussion

In this study we describe for the first time the differentiation of hOMSC- neural crest-like SC isolated from the lamina propria of the human adult oral mucosa into dopamine-secreting cells capable of engrafting in the striatum and improving the symptoms in a rat model of PD. hOMSC are a recently described SC population that exhibits a primitive NCSC-like phenotype [13]. In the present study, we demonstrated that hOMSC respond to developmental cues by differentiating into dopamine-secreting cells with a dopaminergic-like phenotype.

Similarly to other cell types [28]–[31], naive hOMSC were positive for neuronal and dopaminergic markers. Some of these markers as for example Nurr1 and Lmx1A were localized within the cytoplasm of these naïve cells. Several recent studies have shown that the cytoplasmic localization of transcription factors in non-dopaminergic cells is associated with cellular functions (e.g. migration and proliferation) that are unrelated to the development and maintenance of the dopaminergic phenotype [32]–[35]. Since the majority of the naïve hOMSC were positive for dopaminergic markers, the efficiency of the differentiation protocol was evaluated at the molecular level by testing the change in the expression of dopaminergic and neuronal genes and at the cellular level by determining the increase in the number of differentiated hOMSC that exhibited nuclear localization of dopaminergic transcription factors.

Notably, in contrast to LmxA1 and Nurr1, Foxa2, which is considered a pivotal transcription factor for dopaminergic specification, was localized only to the nucleus of naïve hOMSC [19]. To the best of our knowledge this transcription factor is not expressed constitutively by other adult mesenchymal derived stem cells. As Foxa2 plays a role in the development of the ventral midbrain and since the lamina propria of the oral mucosa originates from the mesencephalic neural crest [36] the constitutive nuclear expression and localization of Foxa2 in hOMSC might suggest a certain level of epigenetic memory that render this population more prone to differentiate along the dopaminergic lineage. The design of our differentiation protocol was based on these findings and on the previous characterization of hOMSC as a primitive neural crest stem cell population [13]. FoxA2 is a marker of the neural floor plate and a key transcription factor that controls the specification and differentiation of DA neurons [19]. The designed protocol includes the use of Shh, Wnt-1, FGF-8 and ascorbic acid. The selection of these soluble factors was based on the following data: i) in FoxA2 positive cells, Shh enhances Lmx1A [21], an important transcription factor in the development of the DA phenotype [37]–[41]; ii) Wnt1 is involved in DA progenitor specification and final differentiation. Moreover, Lmx1A in combination with Wnt-1 induce the expression of Pitx3 and Nurr1 that are involved in the final differentiation and function of the DA phenotype [36], [42]–[46]; and iii) FGF-8 acts synergistically with Shh to strengthen the DA phenotype in Nurr1 positive cells [47] and FGF-8 and ascorbic acid, reinforce the generation of TH positive cells during ESC dopaminergic differentiation [48].

This differentiation protocol induced in the differentiated hOMSC changes at the molecular and protein levels as evidenced by: i) a decrease in the pluripotency associated factors; ii) an increase in the expression of neuronal and early and late dopaminergic genes as assessed by quantitative RT-PCR; iii) an increase in the nuclear localization of dopaminergic transcription factors as detected by quantitative immunofluorescence; and iv) a substantial increase in the number of cells positive for both neuronal and dopaminergic markers.

The differentiation process rendered 77% of differentiated hOMSC positive for both βIII-tubulin and tyrosine hydroxylase indicating that the large majority of the population was neuronal and dopaminergic-like in nature. The finding that this population release dopamine upon KCl challenging indicates that this transmitter is synthesized in induced hOMSC. Dopamine release by KCl is an accepted method for evaluating dopamine synthesis *in vitro*. This release is likely to occur due to depolarization of the membrane and consequent calcium entry caused by the activation of voltage dependent calcium channels, since inhibitors of this channels block dopamine release [49]. This approach has been used for demonstrating dopamine release of striatal primary cultured cells [50] and PC12 cells [49] or following dopaminergic differentiation of several SC sources such as embryonic SC [24], mesenchymal SC [51], mesencephalic precursors [52], iPS [53] and induced DA neurons [11]. In the context of these studies, KCl induced-dopamine release by hOMSC-DA demonstrates that these cells are able to synthesize and release dopamine, and therefore they may be endowed with therapeutic activity.

To test this hypothesis the functional and therapeutic potential of hOMSC-DA were tested in the model of 6OHDA hemiparkinsonian rat [54]–[58]. The reduction (approximately 50%) in amphetamine-induced rotations obtained with hOMSC-DA during the experimental period are in the range of those obtained by transplantation of DA neurons derived from reprogramed fibroblasts [10], ESC [59] or IPS derived from PD patients [60]. A trend in rotation reduction was observed in rats treated with naïve hOMSC. This trend reached statistical significance compared to the group of rats treated with saline only at 8 weeks after transplantation. This modest improvement in the rotation test might be explained by the constitutive capacity of naïve hOMSC to secrete neurotrophic factors such as FGF-2, VEGF, EGF and NGF [13]. Since administration of 6OHDA in the *medial forebrain bundle* causes gradual death of dopaminergic neurons [58], it is conceivable that neurotrophic factors secretion into the striatum, specifically in the proximity of axon terminals, could lead by retrograde transport to the survival signaling cascade in damaged, but not dead, DA neurons of the *substantia nigra.*

HPLC analysis demonstrates an increase in the dopamine content of injured hemispheres transplanted with hOMSC-DA as compared to the hemispheres treated with saline or naïve hOMSC. As the HPLC assay does not differentiate between intracellular and extracellular dopamine, the level of dopamine in the hOMSC-DA treated hemispheres does not prove that dopamine is released into the striatum by hOMSC-DA. However, considering the relative higher level of dopamine in the hOMSC-DA treated hemispheres and the significant improvement in the motor function of the hOMSC-DA treated animals compared to the animals in the control groups, it is conceivable to assume that dopamine is released into the striatum of the experimental animals. The mechanisms responsible for this possible release remains to be elucidated in future studies.

Taken together, the results of the present study and the fact that oral mucosa is a readily accessible source for the generation of trillions of hOMSC [13] point to the possibility that hOMSC population might serve for future cell therapy in PD. When treated with dexamethasone and implanted as aggregates on fibrin membranes under the skin, hOMSC develop into a teratoma-like structure consisting of tissues derived from the NC [13]. However, in spite of this safety concern, when dexamethasone treatment is omitted or the cells are implanted as cell suspensions, no such teratoma-like structures ever developed [13], [61]. Within the limitations of the present study, tumor formation was not observed in any of the transplanted brains, suggesting that intracranial transplantation of naïve or differentiated hOMSC might be safe as far as tumorogenesis is concerned. Moreover, to achieve the endeavor of hOMSC therapy in PD a number of issues remain to be elucidated in further studies: i) it is unknown for how long the dopaminergic phenotype of hOMSC-DA will remain stable at the implantation site; ii) the hOMSC used in the present study were obtained from relatively young and healthy donors. Our preliminary results (data not shown) indicate that donor age does not affect neuronal differentiation. It was recently shown that dopaminergic cells derived from iPS generated from sporadic PD patients, but not familial PD patients, exhibited comparable results regarding differentiation and therapeutic potential [60], [62]. Nevertheless, it is unknown whether hOMSC derived from familial and sporadic PD patients would be endowed with the same propensity to differentiate into DA-like neurons and maintain their phenotype *in vivo* as do the hOMSC-DA derived from young and healthy donors; and iii) hOMSC were transplanted into and maintained their phenotype in the striatum of relative young animals. The majority of PD patients are elderly. Thus, it remains to be established how the age of the host and a “hostile” environment affects the function of hOMSC-DA *in vivo.* Based on hOMSC embryonic origin, their constitutive capacity to express dopaminergic markers, the expression level of which is augmented by developmental soluble factors, the fact that their transplantation significantly improve Parkinsonian-like symptoms in rats, and their readily-accessible niche, our study propose hOMSC as a potential therapeutic source for cell replacement therapy for PD.

## Supporting Information

Figure S1 **Negative controls for immunofluorescence and immunochemistry.** Goat anti-mouse antibodies conjugated to Alexa 568 and goat anti-rabbit antibodies conjugated to Alexa 488 were used as secondary antibodies in all the immunofluorescence assays. Panels A and C illustrate β-III tubulin stained with mouse anti-human primary antibodies and the negative control stained with goat anti-mouse secondary antibodies, respectively; panels B and D illustrate Lmx1A stained with rabbit anti-human primary antibodies and the negative control stained with goat anti-rabbit secondary antibodies, respectively. Cells were stained with DAPI for nuclear detection. Tissue sections from 6-OHDA lesioned rats were stained for TH and developed with DAB (E–F). TH immunodetection was performed by using monoclonal anti-TH antibodies and futher incubated with biotin anti-mouse secondary antibody and streptavidin conjugated horseradish peroxidase (E). Negative control incubated only with anti-mouse secondary antibody and streptavidin conjugated horseradish peroxidase (F).
(TIF)

Figure S2 **Western blot analysis showing antibody-staining specificity.** Western blot analysis of fibroblasts, naïve and differentiated hOMSC using the Lmx1A (A) and Nurr1 (B) antibodies. The obtained results show a unique band at the expected molecular weight for each respective protein (Lmx1A 50 KDa and Nurr1 67 KDa). For internal control purposes the anti-emerin antibody was used (emerin 35 KDa), showing similar total protein levels.
(TIF)

Figure S3 **Induced hOMSC show mature dopaminergic-like phenotype, complete figure.** Immunofluorescence analysis and cell positive counts of neuronal and DA markers in naïve, DA-hOMSC and fibroblasts before and after differentiation, scale bar = 50 µm.
(TIF)

Table S1 **Primer sequences used for RT-PCR analysis of pluripotency, neuronal and dopaminergic markers.**
(DOCX)
