# Supplementary figures and images for: Dopaminergic-Like Neurons Derived from Oral Mucosa Stem Cells by Developmental Cues Improve Symptoms in the Hemi-Parkinsonian Rat Model
Source: PLoS One. 2014 Jun 19;9(6):e100445. doi: 10.1371/journal.pone.0100445 (PMC4063966; doi:10.1371/journal.pone.0100445)

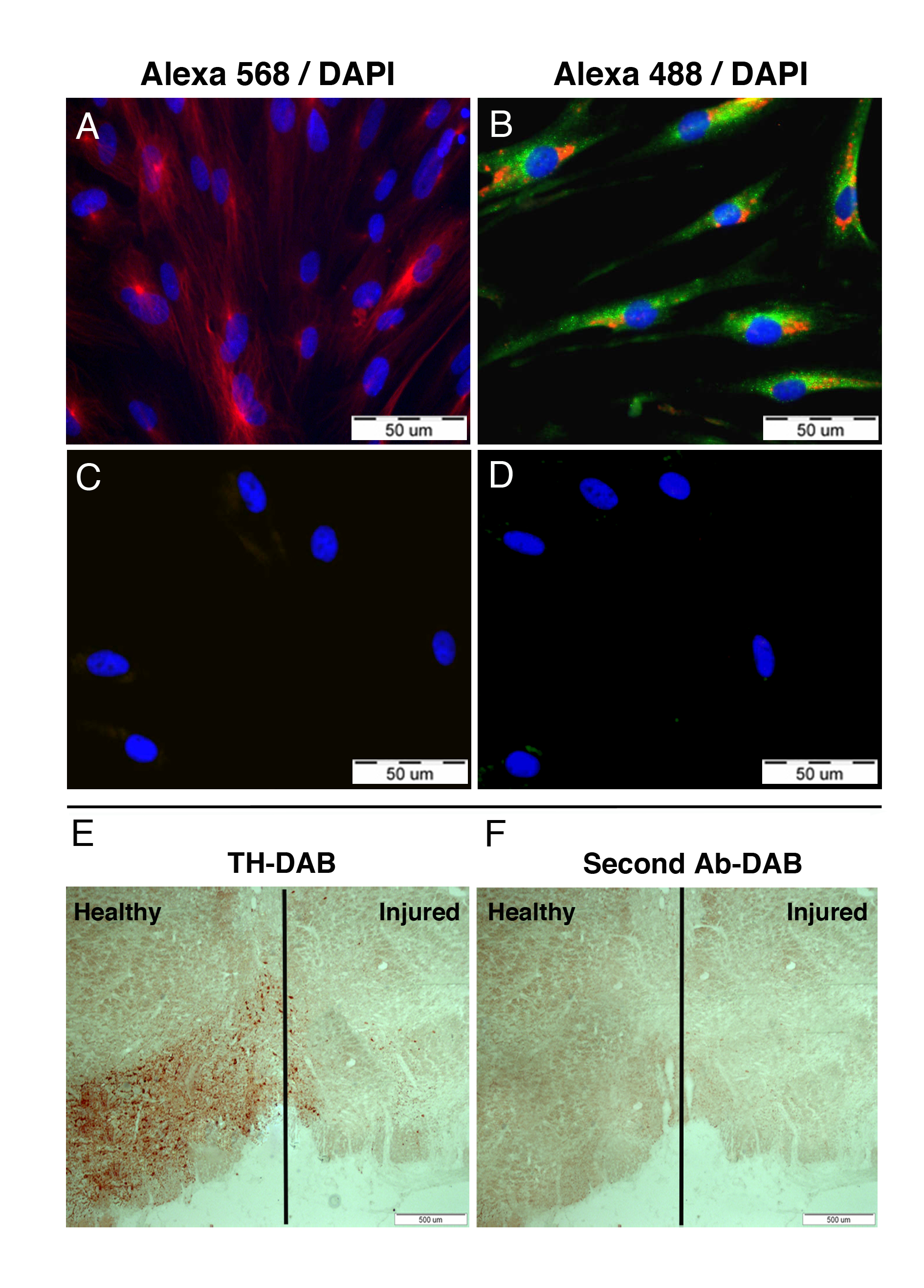

Supplement: Figure S1 — Negative controls for immunofluorescence and immunochemistry. Goat anti-mouse antibodies conjugated to Alexa 568 and goat anti-rabbit antibodies conjugated to Alexa 488 were used as secondary antibodies in all the immunofluorescence assays. Panels A and C illustrate β-III tubulin stained with mouse anti-human primary antibodies and the negative control stained with goat anti-mouse secondary antibodies, respectively; panels B and D illustrate Lmx1A stained with rabbit anti-human primary antibodies and the negative control stained with goat anti-rabbit secondary antibodies, respectively. Cells were stained with DAPI for nuclear detection. Tissue sections from 6-OHDA lesioned rats were stained for TH and developed with DAB (E–F). TH immunodetection was performed by using monoclonal anti-TH antibodies and futher incubated with biotin anti-mouse secondary antibody and streptavidin conjugated horseradish peroxidase (E). Negative control incubated only with anti-mouse secondary antibody and streptavidin conjugated horseradish peroxidase (F). (TIF) [file pone.0100445.s001.tif]

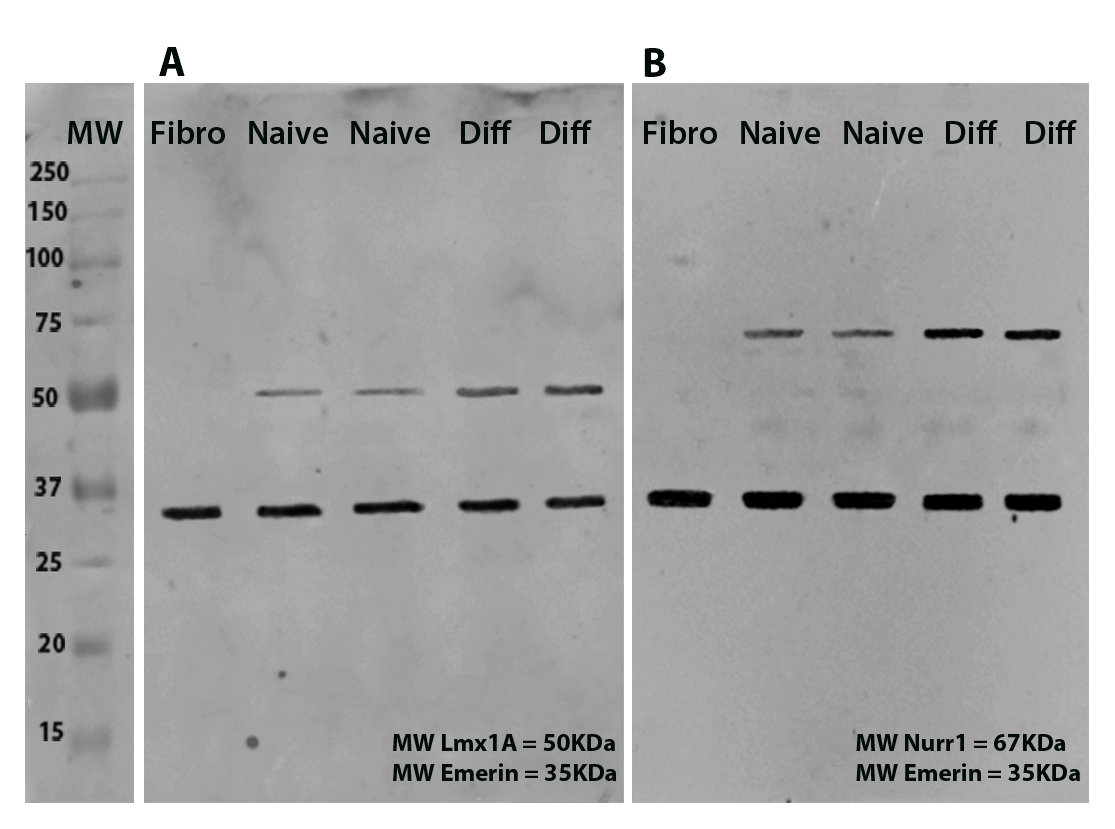

Supplement: Figure S2 — Western blot analysis showing antibody-staining specificity. Western blot analysis of fibroblasts, naïve and differentiated hOMSC using the Lmx1A (A) and Nurr1 (B) antibodies. The obtained results show a unique band at the expected molecular weight for each respective protein (Lmx1A 50 KDa and Nurr1 67 KDa). For internal control purposes the anti-emerin antibody was used (emerin 35 KDa), showing similar total protein levels. (TIF) [file pone.0100445.s002.tif]

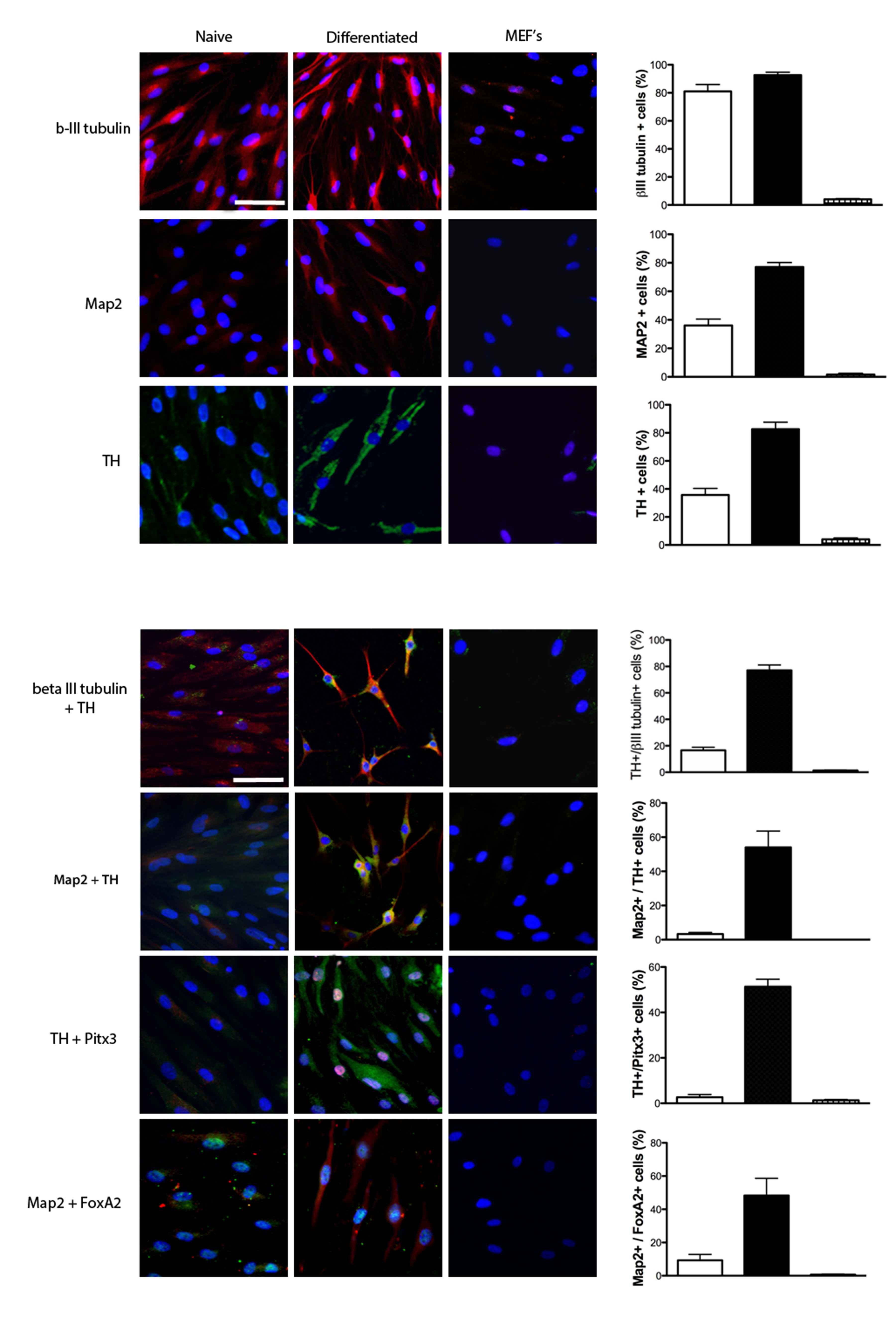

Supplement: Figure S3 — Induced hOMSC show mature dopaminergic-like phenotype, complete figure. Immunofluorescence analysis and cell positive counts of neuronal and DA markers in naïve, DA-hOMSC and fibroblasts before and after differentiation, scale bar = 50 µm. (TIF) [file pone.0100445.s003.tif]
