# Supplementary material for: Dopaminergic-Like Neurons Derived from Oral Mucosa Stem Cells by Developmental Cues Improve Symptoms in the Hemi-Parkinsonian Rat Model
Source: PLoS One. 2014 Jun 19;9(6):e100445. doi: 10.1371/journal.pone.0100445 (PMC4063966; doi:10.1371/journal.pone.0100445)
Supplement: Table S1 — Primer sequences used for RT-PCR analysis of pluripotency, neuronal and dopaminergic markers. (DOCX) [file pone.0100445.s004.docx]

| Primer | Forward | Reverse |
| --- | --- | --- |
| GAPDH | CGACAGTCAGCCGCATCTT | CCAATACGACCAAATCCGTTG |
| Nanog | TGCCTCACACGGAGACTGTC | AGTGGGTTGTTTGCCTTT |
| Sox2 | CAGGAGAACCCCAAGATGC | GCAGCCGCTTAGCCTCG |
| Lmx1a | CCTGCAGGAAGGTGAGAGAGA | TGGACGACACGGACACTCAG |
| Pitx3 | TCTGGAAGGTCGCCTCTAGCT | TCTGGAAGGTCGCCTCTAGCT |
| Otx2 | CGAGGGTGCAGGTATGGTTTA | TCCCGAGCTGGAGATGTCTT |
| DAT | CCAGCAATGACCATGAAG | AGGCCACCCATGAGTAGG |
| Oct4 | CTTCCCTCCAACCAGTTGCCCCAAAC | GACAGGGGGAGGGGAGGAGCTAG |
| FoxA2 | GGGAGCGGTGAAGATGGA | TCATGTTGCTCACGGAGGAGTA |
| NCAM | AACCACTCAGACTACATCTGCCAC | CCTGTCAATCATGCTGTTGGTG |
| FoxA1 | GGCTGAAACCAGCGACTG | AGGCTCCTGCGTGTCTG |
